# Explaining variation in the uptake of HPV vaccination in England

**DOI:** 10.1186/1471-2458-11-172

**Published:** 2011-03-22

**Authors:** Varun M Kumar, David K Whynes

**Affiliations:** 1 Division of Epidemiology and Public Health, University of Nottingham, Nottingham NG5 1PB, UK; 2 School of Economics, University of Nottingham, Nottingham NG7 2RD, UK

## Abstract

**Background:**

In England, two national programmes of HPV vaccination for girls have been instituted, a routine programme for 12- and 13-year-olds and a catch-up programme for 17- and 18-year-olds. Uptake rates across the country have been far from uniform, and this research sought to identify factors explaining the variation in uptake by locality.

**Methods:**

An association between uptake, deprivation and ethnic background had been established in pilot research. The present analysis was conducted at an aggregate, Primary Care Trust (PCT), level for the first year of the programmes. Published measures of HPV vaccination uptake, material deprivation, ethnic composition of PCT populations, primary care quality, and uptake of cervical screening and of other childhood immunisations were collated. Strong evidence of collinearity amongst the explanatory variables required a factor analysis to be undertaken. This provided four independent factors, used thereafter in regression models to explain uptake by PCT.

**Results:**

The factor analysis revealed that ethnic composition was associated with attitudes towards cervical screening and other childhood vaccinations, whilst material deprivation and quality of primary care were orthogonal. Ethnic composition, early childhood vaccination, cervical screening and primary care quality were found to be influential in predicting uptake in both the routine and the catch-up cohorts, although with a lower degree of confidence in the case of the last two independent variables. Lower primary care quality was significant in explaining a greater fall in vaccination uptake between the first two doses in the catch-up cohort. Greater deprivation was a significant explanatory factor for both uptake and the fall in uptake between doses for the catch-up cohort but not for uptake in the routine cohort.

**Conclusion:**

These results for uptake of the first year of the national programme using aggregate data corroborate findings from intentions surveys and pilot studies. Deprivation, the ethnic composition of the population, the effectiveness of primary care and the acceptability of childhood vaccinations are salient factors in explaining local HPV vaccine uptake in England.

## Background

Two types of human papillomavirus (HPV), 16 and 18, have been associated with around 70 per cent of invasive cervical cancers. The virus is transmitted sexually and prophylactic vaccines appear to be efficacious in uninfected women [1]. In England, the Joint Committee on Vaccination and Immunisation [2] evaluated the available scientific evidence [3] and recommended that an HPV vaccination protocol be added to the national childhood immunisation schedule, ostensibly as a cervical cancer preventative. From September, 2008, it became routine for girls aged 12 or 13 years to be offered the bivalent vaccine, Cervarix (GlaxoSmithKlein). Simultaneously, a "catch-up" vaccination programme was initiated for older girls, those aged 17 or 18 years. The arrangements for administering the vaccine across England were devolved to the local Primary Care Trusts (PCTs), most of whom followed the Joint Committee's recommendation in opting for schools-based delivery of the routine programme. Data for the first full year of the scheme indicated that around 88 per cent of girls in England and eligible as a matter of routine received at least one dose of the vaccine, although the variation in uptake by individual PCT was substantial (54 to 100 per cent). The mean first-dose uptake in the catch-up cohort was lower (around 62 per cent), and the variation by PCT was even greater (8 to 97 per cent) [4]. This paper reports a search for factors explaining differences in HPV vaccine uptake by PCT.

Previous studies offer likely contributors to an explanation. The vaccination of children in England is subject to parental approval and surveys of intent conducted prior to the programme's announcement revealed that most parents knew little about HPV, whilst many were concerned about sexual health issues that would arise as a result of a vaccination programme. Acceptability of daughters' HPV vaccination was significantly less likely in non-white households [5,6]. Pilot research in two PCTs in north-west England, conducted prior to the implementation of the main programme, revealed lower uptake in more deprived areas and amongst ethnic minority girls. HPV vaccination was also less likely if other childhood vaccines had not been received [7,8]. Beyond the English evidence, previous vaccination history was predictive of uptake in a Canadian schools programme [9]. Race, religion and deprivation predicted uptake in a catch-up campaign in the Netherlands [10]. In the USA, a study of girls insured through a large managed care organisation identified higher household income, a family experience of sexually-transmitted disease, receipt of other vaccines and mothers' attendance for cervical cancer screening as positive predictors of HPV vaccination [11,12].

## Methods

The intention was to construct linear regression models of uptake for the first 12 months of vaccination, at the level of the PCT. At the time, there were 152 PCTs covering England. Throughout the programme's first year, the average PCT was responsible for the vaccination of around 2,000 girls in the routine cohort and slightly more in the catch-up group. The complete course of HPV vaccination entailed the administration of three doses at 0, 1-2 and 6 months. Although uptake data [4] were nominally available for all three doses, we felt constrained to use uptake to the second dose only as the principal dependent variable. Administrative delays in some of the PCTs had required the administration of the third dose beyond the end of study period, implying that third-dose uptake in those cases was appreciably under-recorded.

Data were obtained for the following explanatory variables:

1) Material deprivation, as measured by the Index of Multiple Deprivation (IMD) for 2007 [13]. The IMD comprises 38 indicators of poverty and deprivation, each calculated individually at the level of the "lower super output area". England is divided into 32,482 such areas and deprivation for any PCT is the summary of deprivation for the output areas which it subsumes. Four routinely-calculated summary statistics were used:

a) The population-weighted mean of the IMD scores for each of the output areas within the PCT. Individual output area scores varied between approximately zero and 85, with a higher score indicating greater deprivation.

b) The population-weighted average of the combined ranks of the IMD scores for the output areas in the PCT (least-deprived output area ranked 1, and most-deprived ranked 32,482).

c) As a measure of the extent of deprivation, the proportion of the PCT's population living in the 10 per cent most deprived output areas in England.

d) As a measure of the local concentration of deprivation, the population-weighted average of the ranks of the PCT's most deprived output areas which contain exactly 10 per cent of the PCT's population.

2) Uptake of routine cytological screening for cervical pre-cancer by PCT for 2008-9, for two age ranges: women aged between 25 and 49 years, and those aged between 50 and 64 years [14]. The uptake of screening is a measure of women's attitudes towards cervical cancer prevention, and the majority of the mothers of children offered HPV vaccination would occupy the younger age range.

3) Uptake of other vaccines in the national childhood immunisation schedule in 2008-9, by PCT [15], namely:

a) Diphtheria, tetanus, pertussis, polio and haemophilius influenzae Type B (DTaP/IPV/Hib) at age 2 years.

b) Measles, mumps, rubella (MMR) and meningitis C (MenC) at age 2 years.

c) Primary doses of diphtheria, tetanus, polio (DT/IPV) at age 5 years.

d) Booster dose of diphtheria, tetanus, pertussis, polio (DTaP/IPV) at age 5 years.

e) MMR first dose (MMR1) and second dose (MMR2) at age 5 years.

4) Prevalence of, and uptake of screening for, other sexually-transmitted infections, represented by PCT-specific rates from the national chlamydia screening programme for those aged between 15 and 24 years [16].

5) Ethnic/racial identity, as measured by the 2007 ethnic composition of the population of each PCT [17]. Proportions were classified as White, Black (African or Caribbean), Asian (the Indian sub-continent only) and Other (of which Chinese was the predominant race).

6) The quality of performance of health professionals in primary care, as measured by summary statistics from the Quality and Outcomes Framework (QOF) for each PCT. The QOF assesses independently four domains for each practice, namely, clinical (the management of specific medical conditions), quality of practice organisation, quality of patient experience and the delivery of additional services [18]. Each domain comprises a set of achievement measures against which practices score points according to performance, and scores are expressed as a proportion of the maximum points achievable.

Given existing research findings, it was anticipated that there would exist a high degree of collinearity between the independent variables. It has already been established, for example, that the parental attitudes and behaviours which predict receipt of primary vaccines at an early age predict subsequent vaccinations equally well [19]. Thus British children un-immunised with primary vaccines at age 1 are at least ten-times less likely to be immunised with MMR at age 3 [20]. The uptake of childhood vaccines has been linked both to deprivation [21,22] and to ethnic composition [23]. Deprivation and ethnic background appear to condition the uptake of all forms of cancer screening [24] and have been associated with cervical screening compliance specifically in studies conducted in the north-west [25] and in the north-east [26] of England. The likelihood of having ever undertaken cervical screening has been shown to be more than twice as high for White women than for women of other races [27]. Cervical screening uptake is a component of the fourth QOF domain (additional services) and deprived areas experience lower quality of care, as assessed by the QOF [28]. Also, deprivation has been linked to both the coverage and the positive rate of chlamydia screening [29]. In view of the collinearities, therefore, a principal components analysis of the explanatory variables was undertaken to produce un-correlated factors, and regression models were constructed from the factor scores of the components identified.

All data used in the analysis were originally compiled by government or official bodies and are openly available. All statistical analyses were conducted using SPSS 16.0.

## Results

Table 1 displays descriptive statistics for all variables. For the routine cohort, serial adherence to HPV vaccination across the two doses was high; the average uptake for the second dose was only 2.3 per cent (SD 3.7) lower than the average uptake for the first. Adherence was lower in the catch-up cohort, however; the corresponding decline in average uptake between the first two doses was 13.8 per cent (SD 10.2). The difference in adherences between the cohorts was significant (t = 13.6, p < 0.01).

**Table 1:** Descriptive statistics for 152 PCTs

	Mean	Standard Deviation	Minimum	Maximum
Number of girls eligible				
Routine	1,996	1,167	367	7,730
Catch-up	2,181	1,828	654	7,788
HPV uptake, first dose only, %				
Routine	87.6	7.5	54.0	100.0
Catch-up	60.2	16.6	7.6	97.3
HPV uptake, 2 doses, %				
Routine	85.6	8.3	52.1	100.0
Catch-up	52.2	16.3	5.5	95.7
Deprivation				
Average IMD score	23.7	9.1	8.1	48.3
Average rank	17,569	5,340	6,177	28,396
Extent, %	23.9	19.2	0.3	81.3
Local concentration	29,001	3,055	19,388	32,434
Cervical cancer screening uptake, %				
Ages 25-49 years	72.2	4.7	57.9	81.2
Ages 50-64 years	79.3	3.1	67.8	85.2
Childhood immunisation uptake, %				
DT/IPV, age 5	92.7	6.4	61.5	98.8
DTaP/IPV, age 5	79.8	13.9	21.3	95.2
MMR1, age 5	89.2	5.8	60.2	96.6
MMR2, age 5	78.0	10.8	32.0	91.5
DTaP/IPV/Hib, age 2	94.0	4.9	64.0	99.4
MMR, age 2	84.9	6.4	56.3	94.7
MenC, age 2	92.0	7.4	56.3	98.8
Chlamydia screening, %				
Screening uptake	16.4	5.1	4.2	35.8
Positive test results	7.4	2.0	3.5	12.8
Ethnic composition of population, %				
White	86.8	12.7	35.9	97.9
Asian	7.0	7.8	0.9	43.7
Black	4.2	5.2	0.5	24.1
Other	2.1	1.6	0.6	8.8
QOF scores, %				
Clinical	97.8	1.3	90.9	99.5
Organisation	95.8	2.7	84.1	99.1
Patient experience	83.8	6.3	47.2	94.4
Additional services	97.2	3.2	83.4	100.0

A preliminary factor analysis of the explanatory variables produced a Kaiser-Meyer-Olkin measure of sampling adequacy of 0.81, thereby confirming the expected high degree of inter-correlation within the data. All individual communalities were in excess of 0.60, with the exception of cervical screening in the higher age range (0.45) and the uptake of chlamydia screening (0.25). These two variables were accordingly excluded from the second stage of data reduction, the results of which appear in Table 2. The four principal components explained 81.9 per cent of the total variance. The factor loadings of the individual components, PC1 through PC4, indicate very strong correlations within particular classes of variable, specifically, the four summary statistics for deprivation, the four QOF domains and the seven uptakes of other childhood vaccines. By way of interpretation, the factor PC4 evidently represents the quality of primary care, as assessed by the four QOF criteria. PC2 embodies the four measures of multiple deprivation which are, in turn, associated with a known prevalence of a sexually transmitted disease. PC1 is dominated by the uptake of the other childhood vaccines, which are themselves associated with race and participation in cytological screening. PC3 associates race with cytological screening attendance and the prevalence of sexually transmitted disease. In the two components where ethnic variables appear (PCs 1 and 3), White has the opposite sign to the three non-White ethnicities. In themselves, these two components together explained almost half of the variance in the data.

**Table 2:** Principal components analysis, varimax rotation, loadings > 0.4

	Communalities	PC1	PC2	PC3	PC4
Deprivation					
Average IMD score	0.96		0.94		
Average rank	0.94		0.92		
Extent, %	0.93		0.93		
Local concentration	0.83		0.90		
Cervical screening, ages 25-49 year	0.74	0.43		0.55	
Childhood immunisation					
DT/IPV, age 5	0.82	0.83			
DTaP/IPV, age 5	0.88	0.89			
MMR1, age 5	0.89	0.92			
MMR2, age 5	0.89	0.91			
DTaP/IPV/Hib, age 2	0.85	0.89			
MMR, age 2	0.87	0.92			
MenC, age 2	0.74	0.83			
Chlamydia: positive test results	0.65		0.55	0.56	
Ethnic composition					
White	0.93	0.42		0.81	
Asian	0.82			-0.85	
Black	0.79	-0.67		-0.51	
Other	0.74	-0.50		-0.62	
QOF scores					
QOF Clinical	0.58				0.61
QOF Organisation	0.83				0.91
QOF Patient experience	0.66				0.73
QOF Additional services	0.84				0.76
*% of variance explained*		*32.7*	*19.9*	*15.4*	*13.9*

We constructed regression models explaining two-dose uptakes for the routine cohort, for the catch-up cohort and for the proportionate fall in uptake (non-adherence) between the first and second doses in the catch-up cohort. Each model employed the factor scores of the four principal components as independent variables. In view of the wide variation in numbers eligible for vaccination by PCT (Table 1), an additional size variable was included in the models initially, to test for a scale effect on uptake. For all models, however, the coefficient on numbers eligible emerged as insignificant (at p = 0.17 or greater) and the variable was removed. Table 3 displays the final regression results. PC1 (race/childhood vaccination/cervical screening) was evidently influential in explaining all three of the dependent variables. PC3 (race/screening) and PC4 (primary care quality) were significant predictors of uptake in both the routine and the catch-up cohorts, although with a lower degree of confidence in the case of the last two. Lower primary care quality (PC4) was significant in explaining low adherence in the catch-up cohort. PC2 (multiple deprivation) was a significant explanatory factor for both uptake and fall in uptake for the catch-up cohort but not for uptake in the routine cohort.

**Table 3:** Regression results

	Constant	PC1	PC2	PC3	PC4	**Adjusted r**^**2**^
Uptake at second dose, routine cohort, %						
β	85.60	2.48	-0.27	3.11	1.71	0.25
95% CIs	84.45	1.33	-1.42	1.96	0.06	
	86.75	3.63	0.88	4.26	2.86	
Uptake at second dose, catch-up cohort, %						
β	52.17	3.07	-2.76	2.27	2.27	0.08
95% CIs	49.67	0.56	-5.27	-0.02	-0.25	
	54.68	5.59	-0.24	4.79	4.78	
Fall in uptake between dose 1 and dose 2, catch-up cohort, %						
β	13.75	-1.58	1.82	-1.1	-2.71	0.12
95% CIs	12.22	-3.12	0.28	-2.64	-4.25	
	15.28	-0.05	3.35	0.44	-1.18	

## Discussion

Ethnicity, deprivation, the quality of primary care and the uptake of other childhood vaccinations are evidently salient factors in explaining HPV vaccine uptake at the PCT level. Our aggregate-data results are consistent with those of existing survey research based on the responses of individuals. For example, whilst being more likely to oppose HPV vaccination in children, Asian families have been shown to be more supportive than other non-Whites of other childhood immunisations [30]. This effect can be observed in PC1. On the other hand, the loadings in PC3 suggests that Asian families might well have considered the HPV vaccine in different terms. In the intention studies, Asian parents were more likely to cite sex-related concerns, particularly the potential encouragement of pre-mature or pre-marital sexual relations, as obstacles to HPV acceptance [6]. Such concerns, possibly fuelled by the print media's focus on such matters [31], would not exist in the case of other childhood vaccines.

The regression analysis reveals that ethnic composition dominated the explanation of uptake for the routine cohort and that deprivation became significant only in relation to the catch-up cohort, where uptake was considerably lower. It is probable that the mode of delivery is the root cause of this disparity. For the routine cohort, 94.2 per cent of eligible girls received vaccinations at their schools, yet only 31.4 per cent of girls in the catch-up cohort did so [4]. Most of the catch-up girls were vaccinated in primary care (community clinics or general practice). In a primary care delivery programme, conscious efforts on the part of the girls and their parents need to be made in order to arrange and secure a vaccination appointment. In contrast, an organised vaccination programme conducted during normal school hours reduces the need for personal effort to virtually zero. The decline in serial adherence can be explained, at least in part, as sequential doses representing constant costs at falling marginal benefit, although it appears that such behaviour can be offset, in some degree, by higher-quality primary care. Whether or not physicians consider themselves entitled to offer vaccination in the face of parental disapproval, however, remains moot [32].

The relatively low coefficients of determination indicate that much of the variation in uptake remains to be explained, and it is probable that such explanations lie beyond the scope of aggregate data. For example, the advantage of a school-operated initiative is logistical (established channels of information, peer reinforcement and large numbers of girls in the same place at the same time). However, absences from school on the appropriate dates, owing to illness, for example, annul the method's advantage. Thus, something as simple and random as a local influenza epidemic would be enough to reduce uptake significantly. There are, moreover, reasons why vaccination might be refused over and above the correlates chosen here. The HPV vaccine is relatively new and long-term efficacy and safety remain to be established. As a result, projected benefits and harms from vaccination are subject to considerable uncertainty [33]. The adoption of mass vaccination has been rapid, and there may be suspicions that commercial interests have dominated health concerns in this respect [34]. Reports of adverse side effects, such as pain, swelling, nausea and light-headedness, might disincline other girls to attend. In a Scottish implementation study, around one-quarter of girls reported such side effects, although the main reasons for declining vaccination were inadequate evidence and the lack of any perceived need for protection, given the existing cytology programme [35].

This study has limitations. First, our measure of vaccine uptake is deficient in that it pertains to the first two doses only, as opposed to the full course of three doses. On the one hand, serial adherence in the routine sample across the first two doses proved to be very high, and it is tempting to conclude that this pattern would have continued to completion. On the other, the lower adherence for the catch-up cohort suggests that three-dose completion rates are far less easily predicted from our two-dose uptake rates. This having been said, it seems improbable that the factors explaining low adherence across the first two doses would be irrelevant in explaining similarly-low adherence across all three, were it to have occurred. This issue will, of course, be resolvable once subsequent data are collected and published. Second, the choice of explanatory variables was constrained by the non-availability of data mapped to PCT boundaries, although additional data would probably assist explanation. A variable for religious affiliation, for example, might better illuminate the character of the racial differences identified. Third, the QOF represents, at best, a proxy for organisation quality and further detail on individual practices would doubtless prove enlightening. Finally, it is probable that the aggregate data conceal variation even at the local level. The average PCT, for example, would be assigned over twenty separate schools. A low routine uptake figure, therefore, could be uniform across all the PCT's schools or caused by especially poor and atypical performances in a small minority.

## Conclusions

Our results indicate that high cervical screening attendances, high uptakes of other childhood vaccinations and proportionately large White populations pre-disposed PCTs towards achieving high HPV vaccination uptakes in both routine and catch-up cohorts. Material deprivation, acting independently of race, was influential in explaining a lower uptake rate, and a lower serial adherence, for the catch-up cohort. The quality of primary care was instrumental in explaining both uptakes and adherence and was evidently orthogonal to the other variables. Thus, there was no evidence to suggest that care quality was inferior in PCTs recording greater deprivation. Testing positive for chlamydia negatively influenced uptake when associated with deprivation but positively influenced uptake when associated with cervical screening participation and being White.

## Competing interests

The authors declare that they have no competing interests.

## Authors' contributions

VMK conceived the study, identified the relevant research literature and data, and undertook the preliminary analysis. DKW refined the statistical analysis and drafted the manuscript. Both authors read and approved the final manuscript.

## Pre-publication history

The pre-publication history for this paper can be accessed here:

http://www.biomedcentral.com/1471-2458/11/172/prepub
